# Analysis of the TCGA Dataset Reveals that Subsites of Laryngeal Squamous Cell Carcinoma Are Molecularly Distinct

**DOI:** 10.3390/cancers13010105

**Published:** 2020-12-31

**Authors:** Alana Sorgini, Hugh Andrew Jinwook Kim, Peter Y. F. Zeng, Mushfiq Hassan Shaikh, Neil Mundi, Farhad Ghasemi, Eric Di Gravio, Halema Khan, Danielle MacNeil, Mohammed Imran Khan, Adrian Mendez, John Yoo, Kevin Fung, Pencilla Lang, David A. Palma, Joe S. Mymryk, John W. Barrett, Krupal B. Patel, Paul C. Boutros, Anthony C. Nichols

**Affiliations:** 1 Department of Otolaryngology, Head and Neck Surgery, University of Western Ontario, London, ON N6A 5W9, Canada; asorgini@uwo.ca (A.S.); hkim2022@meds.uwo.ca (H.A.J.K.); yzeng2023@meds.uwo.ca (P.Y.F.Z.); mushfiq.shaikh@lhsc.on.ca (M.H.S.); nmundi2@uwo.ca (N.M.); edigravio2021@meds.uwo.ca (E.D.G.); Halema.Khan@lhsc.on.ca (H.K.); Danielle.Macneil@lhsc.on.ca (D.M.); mkhan953@uwo.ca (M.I.K.); Adrian.Mendez@lhsc.on.ca (A.M.); John.Yoo@lhsc.on.ca (J.Y.); Kevin.Fung@lhsc.on.ca (K.F.); David.Palma@lhsc.on.ca (D.A.P.); jmymryk@uwo.ca (J.S.M.); John.Barrett@lhsc.on.ca (J.W.B.); 2 Department of General Surgery, University of Western Ontario, London, ON N6A 5C5, Canada; Farhad.Ghasemi@lhsc.on.ca; 3 Department of Oncology, University of Western Ontario, London, ON N6A 5W9, Canada; Pencilla.Lang@lhsc.on.ca; 4 Department of Microbiology & Immunology, University of Western Ontario, London, ON N6A 5C1, Canada; 5 Department of Otolaryngology, Moffitt Cancer Center, Tampa, FL 33612, USA; Krupal.Patel@moffitt.org; 6 Department of Human Genetics, University of California, Los Angeles, CA 90095, USA; pboutros@mednet.ucla.edu; 7 Department of Urology, University of California, Los Angeles, CA 90095, USA; 8 Eli and Edythe Broad Center of Regenerative Medicine and Stem Cell Research, University of California, Los Angeles, CA 90095, USA; 9 Institute for Precision Health, University of California, Los Angeles, CA 90095, USA; 10 Jonsson Comprehensive Cancer Centre, University of California, Los Angeles, CA 90095, USA

**Keywords:** genomics, mutational status, larynx cancer, HNSCC, HPV-negative

## Abstract

**Simple Summary:**

Squamous cell carcinomas from different parts of the larynx have distinct presentations and prognoses, but the molecular basis for this discrepancy has yet to be characterized. We aimed to determine whether different types of mutations at the DNA, mRNA, and protein levels exist to explain the differential prognoses observed. We found that cancers of the supraglottis had higher overall and smoking-associated genome mutations. Further, supraglottic cancers had a significantly poorer prognosis when other clinical variables and mutational status were controlled for. Different protein pathways were enriched in each subsite: muscle-related in the glottis and neural in the supraglottis. Specific cancer-related proteins were also differentially abundant between the supraglottis and glottis. Our findings may partially explain therapeutic response differences, but further study is required for validation.

**Abstract:**

Laryngeal squamous cell carcinoma (LSCC) from different subsites have distinct presentations and prognosis. In this study, we carried out a multiomic comparison of LSCC subsites. The Cancer Genome Atlas (TCGA) LSCC cohort was analyzed in the R statistical environment for differences between supraglottic and glottic cancers in single nucleotide variations (SNVs), copy number alterations (CNAs), mRNA abundance, protein abundance, pathway overrepresentation, tumor microenvironment (TME), hypoxia status, and patient outcome. Supraglottic cancers had significantly higher overall and smoking-associated SNV mutational load. Pathway analysis revealed upregulation of muscle related pathways in glottic cancer and neural pathways in supraglottic cancer. Proteins involved in cancer relevant signaling pathways including PI3K/Akt/mTOR, the cell cycle, and PDL1 were differentially abundant between subsites. Glottic and supraglottic tumors have different molecular profiles, which may partially account for differences in presentation and response to therapy.

## 1. Introduction

Laryngeal squamous cell carcinoma (LSCC) is a common form of head and neck squamous cell carcinoma (HNSCC), comprising 30% of all cases [1,2]. LSCC affects three subsites: the supraglottis, glottis and subglottis. The majority of tumors arise in the glottis and supraglottis, while tumors of the subglottis are rare and only comprise 2% of LSCC cases [3,4,5]. Tumors in these different sites are associated with distinct symptoms and stages at presentation, rates of nodal metastases, tobacco exposure and survival [3,4]. Anatomic differences explain a portion of these findings. For example, the supraglottis has a rich lymphatic drainage, while the glottis, derived from a different pharyngeal arch and pouch [6], has a paucity of lymphatic vessels, consistent with the lower rate of lymphatic metastases [3,4]. The anatomical location of the glottis contributes to frequent clinical findings of hoarseness, airway obstruction and cartilage erosion associated with cancers at this subsite [3,4].

The primary subsite also affects the prognosis, with most reports demonstrating that patients with supraglottic LSCC have poorer prognoses compared to those with glottic tumors, while those with subglottic tumors experience an intermediate rate of survival [3,5,7,8]. Although the anatomical factors described above are likely to play a part in these differences, it is unclear if there are molecular underpinnings that also contribute to clinical outcomes. Previous studies have identified molecular differences that are prognostic in LSCC; for example, mutations in the genes NSD1 and NSD2 confer a positive prognostic impact on LSCC (see [9] and refs). However, the nature of the molecular differences between subsites within the larynx and how they impact prognoses remain completely unexplored. We sought to determine whether molecular differences exist between subsites in LSCC, using The Cancer Genome Atlas (TCGA) LSCC cohort. Supraglottic and glottic subsites were compared for differences in the frequency/abundance of single nucleotide variations (SNV), copy number alterations (CNA), transcriptome, proteome, tumor microenvironment (TME) landscape and hypoxia status.

## 2. Results

### 2.1. Clinical Characteristics Differ by Laryngeal Subsite

After reviewing pathology reports, 117 TCGA LSCC patients were classified as 52 supraglottic, 46 glottic, 15 hypopharyngeal and 2 subglottic cancers. Two patients lacked available pathology reports. The subglottic tumors were excluded due to insufficient cases for comparison. The hypopharyngeal tumors were excluded as they are not LSCC. The remaining 98 tumors were screened for HPV status using RNA sequence data for viral transcripts [10,11]. One tumor was identified as HPV-positive and was excluded because HPV-positive cancer is distinct from HPV-negative cancer [12,13,14,15,16,17,18]. Two samples without available HPV data were also excluded. The remaining 95 HPV-negative primary LSCC samples, including 49 supraglottic and 46 glottic tumors, were used for our analyses. Upon univariate analysis, it was found that the glottic cancers were significantly more likely to be category T4 (74% vs. 26%, *p* < 10^−4^) and demonstrate thyroid or cricoid cartilage invasion (79% vs. 21%, *p* < 10^−6^). No significant differences were noted between subsites for age, sex, smoking history, N-category, overall stage or use of adjuvant radiotherapy (Table 1). Between the supraglottis and glottis, no significant differences were noted in overall survival (Figure S1).

### 2.2. Overall SNVs and Smoking-Associated C>T Transversions Were More Frequent in the Supraglottis

**Figure 1:**
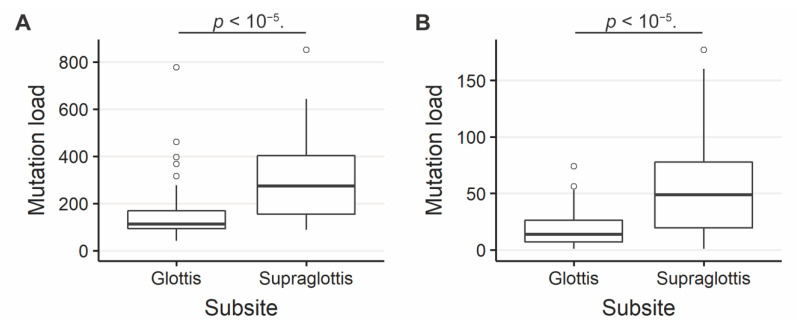
(**A**) Overall SNV mutation load in supraglottic and glottic LSCC. Glottis median value 113.0, interquartile range (IQR) 76.0; supraglottis median 273.0, IQR 247.5. (**B**) G>T SNV mutation load in supraglottic and glottic LSCC. Glottis median 13.50, IQR 19.00; supraglottis median 48.5, IQR 58.25. The number of SNVs per tumor differs significantly between supraglottic and glottic LSCC (*p* < 10^−5^).

**Figure 2:**
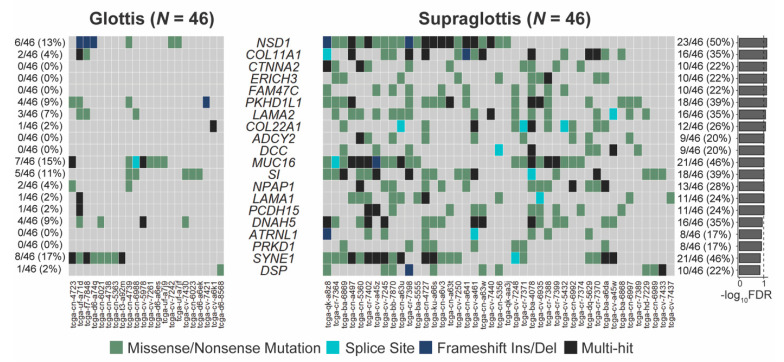
Genes with differing SNV mutation frequencies between supraglottic and glottic LSCC by total SNV mutational load. FDR = false discovery rate.

The SNV mutational load was reported from exome sequencing per tumor as previously described [19,20]. Analysis of exome sequencing data revealed that the total SNV mutational load was significantly higher for supraglottic compared to glottic cancers (glottis: median 113.0, interquartile range (IQR) 76.0; supraglottis: median 273.0, IQR 247.5, *p* < 10^−5^, Figure 1A). Fifteen genes demonstrated more frequent SNVs in the supraglottic cancers (FDR < 0.1, Figure 2). The genes with the most SNV mutations overall within the cohort can be found in Figure S2. NSD1 SNVs demonstrated the greatest differential mutation rate, with 50% of supraglottic LSCC cases harboring alterations versus only 13% in the glottis (FDR = 0.076). It is widely accepted that G>T transversions in HNSCC are associated with patient smoking history [19,20], so we also specifically analyzed the difference in SNVs of this type between subsites. This revealed a higher overall G>T mutation load in the supraglottis (glottis: median 13.50, IQR 19.00; supraglottis: median 48.5, IQR 58.25, *p* < 10^−5^, Figure 1B). DNAH5 was the only gene found to have a differential rate of G>T transversions, occurring in 20% of supraglottic carcinomas and 0% of glottic LSCC (FDR = 0.043, Figure S3). Analysis of the mutational distribution of DNAH5 SNVs across the gene revealed a pattern suggesting the gene may function as a putative tumor suppressor given the broad distribution of the alterations and absence of hotspot sites [21] (Figure S4).

**Figure 3:**
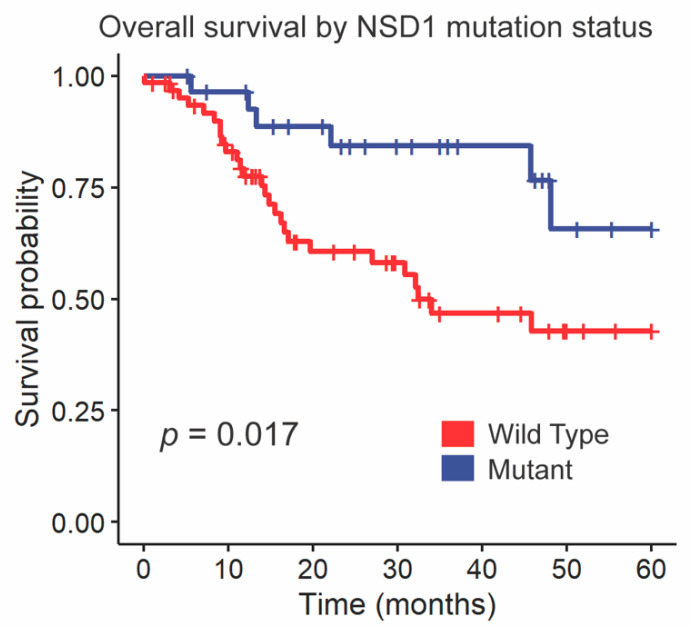
Kaplan-Meier curves for overall survival by *NSD1* SNV mutation status. A log-rank test was used to compare the Kaplan-Meier curves between tumors with wild-type and mutant alleles.

**Figure 4:**
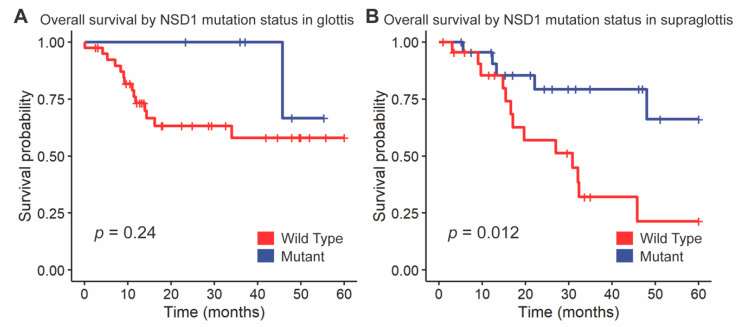
Kaplan-Meier curves for overall survival by *NSD1* mutation status, stratified by (**A**) glottis and (**B**) supraglottis. A log-rank test was used to compare the Kaplan-Meier curves between tumors with wild-type and mutant alleles.

We followed up these findings by investigating the impact of NSD1 and DNAH5 on survival within the HPV-negative cohort of glottic and supraglottic tumors. NSD1-mutant tumors had significantly improved overall survival compared to wild-type tumors overall (*p =* 0.017, Figure 3). When stratified further by subsite, NSD1-mutant tumors were found to not be a prognostic indicator within the glottic cohort (*p =* 0.24, Figure 4A) but were found to have positive prognostic value in the supraglottic cohort (*p =* 0.012, Figure 4B). DNAH5 mutation did not have a significant impact on survival (*p =* 0.41, Figure S5).

There were no differences in CNAs between glottic and supraglottic cancers [22].

### 2.3. Signatures by Subsite Analysis

The top two gene signatures identified in the glottis were COSMIC_13—APOBEC Cytidine Deaminase (C>G) [cosine-similarity: 0.919] and COSMIC_5—Unknown [cosine-similarity: 0.771]. The top two in the supraglottis were COSMIC_4—exposure to tobacco (smoking) mutagens [cosine-similarity: 0.947] and COSMIC_13—APOBEC Cytidine Deaminase (C>G) [cosine-similarity: 0.757 [22].

### 2.4. Multivariate Analysis of Survival with Clinical Variables and NSD1 Mutation Status

**Figure 5:**
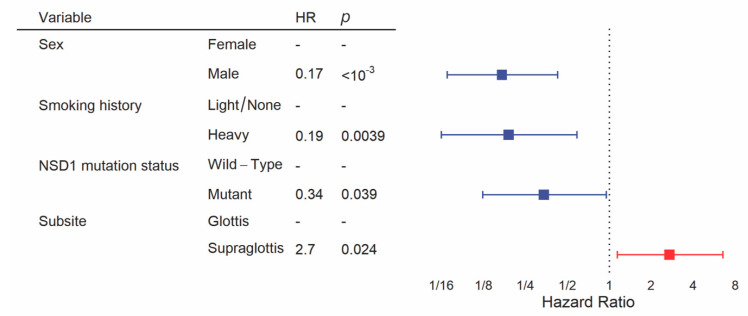
Multivariate analysis of overall survival between glottic and supraglottic LSCC. Backward selection was used to attain the multivariate model. All patients were included into the multivariate model but some were excluded by the algorithm if there were missing values for variables.

We constructed the multivariate model starting with clinical variables and NSD1 mutation status, as we have done previously [19]. Backward selection included sex, smoking history, subsite and NSD1 mutation status in the final model for overall survival. This revealed a significant difference in overall survival between subsites after controlling for other clinical variables and NSD1 mutation status, with significantly poorer prognosis in the supraglottis (HR = 2.7, 95% CI = 1.1–6.5, *p* = 0.024, Figure 5, Table S1). In addition, male sex (HR = 0.17, 95% CI = 0.069–0.43, *p* < 10^−3^), heavy smoking history (HR = 0.19, 95% CI = 0.063–0.59, *p =* 0.039) and NSD1 mutation (HR = 0.34, 95% CI = 0.12–0.95, *p =* 0.0039) were favorably prognostic within this multivariate analysis.

### 2.5. Muscle Contraction and Neural Pathways Were Upregulated in the Glottis

A mRNA-sequencing analysis comparing transcript levels between glottic and supraglottic cancers revealed 122 genes with significantly higher abundance in the former and 160 genes with significantly higher abundance in the latter (FDR < 0.01 and absolute value of the fold change >2, Figure S6, Table S2). Combined Reactome analysis with significant SNVs (*n* = 15) and CNAs correlated with mRNAs (*n* = 282) between subsites revealed 17 significantly upregulated pathways in the supraglottis as compared to the glottis, 5 pathways upregulated in the glottis over the supraglottis, and one with equal numbers of genes upregulated as downregulated (FDR < 0.05). The top upregulated pathways involved muscle contraction in the glottis and neuronal systems in the supraglottis (Table S3).

### 2.6. Protein Expression Differed between Subsites

A RPPA analysis revealed 38 differentially abundant proteins between the glottis and supraglottis (Table S4). The top five included MIG6 (FDR = 0.044), PREX1 (FDR = 0.044), IRS1 (FDR = 0.073), ETS1 (FDR = 0.073), and CHEK1 (FDR = 0.073, Figure S7). None of the corresponding mRNAs were differentially abundant between the glottic and supraglottic cancers, and none of the proteins were significantly associated with patient survival [22]. On Spearman correlation, MIG6 (ρ = 0.27, FDR = 0.046), PREX1 (ρ = 0.41, FDR = 0.0028), IRS1 (ρ = 0.26, FDR = 0.046) and ETS1 (ρ = 0.40, FDR = 0.0028) had significant correlation between protein and mRNA levels (Figure S8). Of note, cancer related proteins RB1, CHEK2, CCNE1, S6 and PIK3CA were higher in the supraglottic cancers, whereas PDL1 was higher in the glottic cancers (Table S4).

### 2.7. CNAs, TME, and Hypoxia Status Did Not Differ between Subsites

Hypoxia analysis revealed no difference in Buffa intratumor type scores between subsites [22]. There were no differences in TME between glottic and supraglottic cancers [22].

## 3. Discussion

Significant differences at the SNV, transcriptome and protein level between supraglottic and glottic cancers were observed. Integrated analysis of molecular data identified differentially activated pathways between the subsites involved with neuronal signaling and muscular contraction. These molecular differences may account for some of the differences in clinical behavior observed between supraglottic and glottic cancers.

In contrast to the genome-wide assays at the DNA and RNA level, the RPPA protein assays were limited to 237 cancer-related proteins and phosphoproteins. While the small number of targets limits the opportunity for discovery, the resulting hits are all canonical cancer signaling proteins and/or potential therapeutic targets. In our analysis, PIK3CA and S6, critical members of the PI3K/Akt pathway [23], were found to be more highly expressed in the supraglottis versus the glottis. Indeed, the PI3K/Akt pathway is the most commonly altered pathway in HNSCC, and its abnormal activation is responsible for tumorigenesis, invasion, metastasis and resistance to anticancer therapy [24]. Cell cycle proteins including CHEK2, CCNE1, and phosphorylated RB1 were expressed at higher levels in the supraglottic cancers, which may indicate a disruption in the G1/S transition. RB1 and CHEK2 act as tumor suppressors, maintaining the cell in G1 [25,26], whereas CCNE1 (cyclin E1) acts as an oncogene when mutated or abnormally activated [27]. Alterations in G1/S transitional control have been previously identified as important in HNSCC [25,28,29]. In combination, the PI3K/Akt pathway and cell cycle alterations may provide a potential mechanism for the higher rates of nodal metastases and poorer survival seen in the supraglottis over other cohorts.

Interestingly, PD-L1 protein levels were significantly higher in glottic cancers. PD-L1 and its receptor PD-1 are critical regulators of the T cell immune response [30,31]. A high level of PD-L1 expression by tumor cells negatively regulates T-cell activation, enhancing evasion of antitumor immunity [30,31]. Interrupting the PD-1 and PD-L1 interaction with immune checkpoint inhibitors (ICIs) has already conferred transformative survival benefits for numerous solid organ malignancies including HNSCC in the recurrent and metastatic setting [32]. Importantly, ICI response is strongly associated with tumor PD-L1 levels [33] suggesting that glottic cancers may be more favorable target for immunotherapy than supraglottic cancers; however, this will need to be confirmed in the context of prospective trials.

The LSCC cohort is associated with a heavier smoking history relative to other head and neck sites, which we have previously linked with higher mutational rates [19]. Here, we identified that even within the larynx, supraglottic cancers have higher overall and smoking-associated mutational loads than the glottic cancers. *NSD1*, which is more frequently altered in the supraglottic cancers, is known to have higher mutation rates in HPV-negative patients with a heavy smoking history [19]. Despite the association with increased tobacco use, tumors harboring this mutation experience a survival benefit in four different HNSCC cohorts, including one limited to laryngeal cancer [9,19,34]. Given that most population-based studies demonstrate poorer outcomes in supraglottic cancer, this is somewhat unexpected. Conversely, we found *DNAH5,* which encodes a protein involved in the dynein complex and functions in ciliary cell motility to have more tobacco associated G>T transversions in the supraglottic cancers [35]. Mutations in *DNAH5* have been associated with poorer survival in a number of different cancers [36,37,38,39]. We did not note an impact of *DNAH5* G>T mutation on survival in this study, but this may reflect the limited sample size of the LSCC cohort. In vitro experiments and prospective cohorts will be required to elucidate the exact role of both *NSD1* and *DNAH5* on LSCC tumor behavior and response to therapy.

We also found that neuronal system pathways were enriched in the supraglottis over the glottis. Interestingly, other researchers have identified that neural related pathways are active in LSCC, including a recent finding of adrenergic nerve fiber involvement in promoting tumor growth is related to loss of TP53 [40]. Interestingly, we did find more TP53 mutations in the supraglottis. Another study found active neural pathways in LSCC involving TrkB, a neurotrophin receptor that regulates neuronal cell proliferation, differentiation and invasion [41]. It is also linked to the epithelial to mesenchymal transition (EMT), a shift in tumor biology characteristic of more aggressive, invasive cancers with worse prognosis [42,43]. Taken together, this may help explain the poorer prognosis seen in the supraglottic versus glottic tumors. Surprisingly, however, muscle contraction pathways which are also linked to the EMT in numerous cancers [44,45,46,47,48], were found to be upregulated in the glottis. This is the opposite to what was expected, but the discrepancy may be related to tissue sampling methods within the HNSCC cohort of the TCGA. While these tumor samples have undergone pathology quality control measures including a purity measure of >60% cellularity [18], samples in the HNSCC cohort have been found to have low tumor purity relative to other TCGA cohorts based on molecular criteria [49]. Therefore, it is plausible that undesired muscular tissue was resected with samples, which may have altered the cell type, and thus, increased the number of pathways that were shown to be significant. More research in this area is needed to determine whether there is a role of muscle contraction pathways in tumorigenesis and invasion.

Several population level studies have demonstrated that supraglottic cancers tend to have poorer survival than glottic cancer [7,50]. In the present study, we also identified a statistically significant difference in survival between supraglottic and glottic cancers using the TCGA LSCC cohort after multivariate analysis. Our findings of the genomic, transcriptomic, proteomic and pathway enrichment differences between subsites suggest that molecular-level differences play important roles in the discrepancies in outcomes between these two subsites.

## 4. Materials and Methods

### 4.1. Data Acquisition

Patient data from TCGA HNSCC dataset, including clinical data, level 3 DNA mutation packager calls data, CNAs, and mRNA abundance were retrieved on June 28, 2019 using latest callsets from The Broad Institute’s Firehose database (version GRCh37) [51,52]. Patient IDs, with larynx primary site, were then identified and used for the analysis. Thereafter, R [53] TCGAbiolinks package [54,55] was used to access the pathology reports for the identified patients also using the Broad Institute’s Firehose database [51,56]. The pathology reports were then reviewed (KBP and AS) to determine the subsite. Subglottic tumors were excluded as there was an insufficient number of cases for comparison (two tumors). HPV status was defined using RNA sequence data for viral transcripts [10,11]. Cartilage invasion was determined as gross invasion of the thyroid or cricoid cartilage only. The T-category, N-category, and overall stages were based on pathologic staging. Buffa et al. TCGA cohort intratumor type hypoxia scores [57] were downloaded from Bhandari et al. Supplemental Material [58]. Patient smoking status was defined as heavy if >20 pack year history based on previous research [19,20], light smoking history was defined as between 0 and 20 pack years, and patients who had never smoked were considered nonsmokers.

### 4.2. Statistics

#### 4.2.1. Clinical Features

In the R statistical environment (version 3.6.1), clinical feature distribution was compared between subsites using Fisher’s Exact Test, Pearson’s X^2^ Goodness of fit test, and Mann-Whitney U test.

#### 4.2.2. Exome Sequence Analysis

Three supraglottic tumors were missing mutational data. Within the R statistical environment (3.6.1) the Bioconductor framework’s maftools package (version 2.0.10) [59] was used to analyze exome sequencing data. Mann-Whitney U tests and Kruskal-Wallis exact tests were used to calculate the differences in total SNV mutation load between subsites, with two-tailed *p* values reported. To identify genes that were differentially mutated between subsites, Fisher’s Exact tests were used. Genes were only evaluated if they were mutated in at least five patients in at least one group. Excluded from the downstream analyses were synonymous mutation variants and the *TTN* gene, as this is one of the longest in the genome and has a high degree of passenger mutations, while it is known to not be an oncogene [60]. The Benjamini-Hochberg adjustment on derived *p* values for false discovery rate (FDR) was set at a threshold of 0.1 for significance [10,19,30]. Signatures were estimated and extracted within the maftools package in R statistical environment. The trinucleotide matrix of the gene sequences for each subsite was first derived, then the number of signatures was estimated as two based on the cophenetic correlation coefficient, thus the matrix was decomposed into two signatures.

#### 4.2.3. Copy Number Alterations

Using data downloaded from GISTIC2 analysis of the TCGA HNSCC cohort, copy number alterations (CNAs) were compared for significant differences in shallow and deep deletions, gains, and amplifications between individual genes. Homozygous losses made up the definition of deep deletions (with GISTIC2 value of −2), whereas heterozygous losses were indicated by shallow deletions (GISTIC2 value of −1). A GISTIC2 value of +1 defined CNA gains and GISTIC2 of +2 defined amplifications. CNA frequency between subsites was compared using Fischer’s Exact tests, and derived *p* values were corrected for FDR as described previously [10,19,30].

#### 4.2.4. mRNA and Pathway Analysis

The DESeq2 package (version 1.24.0) [61] in the R statistical environment was used to normalize and analyze the TCGA LSCC RNA sequencing count data. A negative binomial generalized linear distribution was used to model mRNA abundance profiles for the supraglottic and glottic subsites. Within the DESeq2 package, a Wald test with shrinkage estimation log_2_ fold-change values was used to compare these values. As previously described, FDR adjustment was performed [10,19,30]. Reactome Pathway Analysis [62,63] was performed in the R statistical environment with significant SNVs and significant mRNA-sequence data to analyze for over-representation of pathways.

#### 4.2.5. Reverse Phase Protein Array (RPPA) Analysis

From The Cancer Proteome Atlas [64,65], processed normalized RPPA data were downloaded. Mann-Whitney U tests and FDR corrections [19,30] were used to compare relative protein abundance between groups. Spearman’s rank correlation was used for validation of the differentially expressed proteins with their corresponding mRNA abundance values.

#### 4.2.6. Tumor Microenvironment Estimation

The TCGA RNA-sequence data was analyzed in the immunedeconv package in R (v 2.0.0) to estimate the TME composition of each sample. This package uses the Microenvironment Cell Populations-counter (MCP-counter) method [66] to compute a score. MCP is an accurate method for comparisons between samples [67]. To create the score, it utilizes transcriptomic markers which have previously been demonstrated to be characteristic of the specific immune cell populations and their abundance within the tumor. Mann-Whitney U tests were used to compare intercohort scores for T cells, B cells, natural killer (NK) cells, myeloid dendritic cells (DCs), monocytes, neutrophils, cancer-associated fibroblasts (CAFs), and endothelial cells. As previously described, FDR adjustment was also completed [10,19,30].

#### 4.2.7. Survival Analysis

The R survival package (v 2.44-1.1) [68] was used to perform survival analyses. Between subsites, overall and progression-free interval outcomes were compared using the log-rank test and construction of Kaplan-Meier curves. For univariate and multivariate survival analyses of clinical covariates, the Cox proportional hazards model with the Wald test on individual coefficients was used. These clinical covariates included laryngeal subsite, cartilage invasion status, pathologic T-category, pathologic N-category, overall TNM stage, age, sex, smoking history and treatment with adjuvant radiotherapy. A backward stepwise analysis was used to develop the multivariate model.

#### 4.2.8. Hypoxia

The Supplemental Materials and the scores for intratumor hypoxia were downloaded from Bhandari et al. and Buffa et. al, respectively [57,58,69]. Mann-Whitney U tests with FDR adjustment were used to compare intercohort scores [11,52].

## 5. Conclusions

The present study has demonstrated significant genomic, transcriptomic and proteomic differences between supraglottic and glottic HPV-negative LSCC. Further studies involving larger cohorts treated with uniform treatment paradigms, as well as in vitro functional studies, are needed to understand the impact of these molecular findings regarding therapy and patient survival.

## Figures and Tables

**Table 1:** Clinical characteristics of the TCGA LSCC cohort by primary subsite of cancer.

Variables		HPV Negative Samples, No. (%) (*n =* 95)		
		Glottis (*n =* 46)	Supraglottis (*n =* 49)	*p* Value ^1^
Age	Median (range)	61.5 (47–80)	62 (38–83)	0.783
Sex	Female	6 (13)	13 (27)	0.13
	Male	40 (87)	36 (73)	
Smoking history	Nonsmoker	4 (11)	1 (3)	0.17
	Light smoker	4 (11)	2 (5)	
	Heavy smoker	27 (77)	36 (92)	
T-category	T1	1 (2)	4 (9)	**<10^−4^**
	T2	5 (11)	5 (11)	
	T3	4 (9)	17 (36)	
	T4	34 (74)	12 (26)	
	TX	2 (4)	9 (19)	
N-category	N0	23 (50)	15 (32)	0.084
	N1	7 (15)	5 (11)	
	N2	9 (20)	16 (34)	
	N3	2 (4)	0 (0)	
	NX	5 (11)	11 (23)	
Overall stage	I	1 (2)	1 (3)	0.25
	II	3 (7)	5 (14)	
	III	4 (9)	8 (22)	
	IV	36 (82)	23 (62)	
Adjuvant radiotherapy	no	11 (31)	11 (26)	0.62
	yes	24 (69)	32 (74)	
Cartilage invasion	no	8 (21)	30 (79)	<10^−6^
	yes	31 (79)	8 (21)	

^1^ Bolded *p* values are significant.

## Data Availability

Publicly available datasets were analyzed in this study. This data can be found here: [Firehose, Broad Institute of MIT and Harvard. Available online: https://gdac.broadinstitute.org/ (accessed on 28 June 2019)].

## Supplementary Materials

The following are available online at https://www.mdpi.com/2072-6694/13/1/105/s1, Figure S1: Kaplan-Meier curves for overall survival between supraglottic and glottic LSCC, Figure S2: Top 20 genes by SNV frequency in the combined TCGA LSCC cohort, Figure S3: Genes with differing SNV mutation frequencies between supraglottic and glottic LSCC by G > T mutation load, Figure S4: Lollipop plot of SNVs in *DNAH5,* Figure S5: Kaplan-Meier curves for overall survival by *DNAH5* SNV mutation status Figure S6: Volcano plot for mRNA abundance between glottic and supraglottic LSCC, Figure S7: Differentially abundant proteins between supraglottic and glottic LSCC, Figure S8: Four most differentially abundant proteins between supraglottic and glottic LSCC correlated with mRNA levels. Table S1: Multivariate analysis of overall survival, Table S2: List of genes with significantly different mRNA abundance or SNV frequencies between glottic and supraglottic LSCC, Table S3: Pathway over-representations from genes with differential frequencies of SNVs and CNAs correlated with mRNAs between glottic and supraglottic LSCC, Table S4: RPPA differences between supraglottic and glottic LSCC.
