# Supplementary material for: Analysis of the TCGA Dataset Reveals that Subsites of Laryngeal Squamous Cell Carcinoma Are Molecularly Distinct
Source: Cancers (Basel). 2020 Dec 31;13(1):105. doi: 10.3390/cancers13010105 (PMC7794818; doi:10.3390/cancers13010105)
Supplement: Supplementary file 1 [file cancers-13-00105-s001.pdf]

Supplementary Materials: Analysis of the TCGA Dataset Reveals that Subsites of Laryngeal Squamous Cell Carcinoma are Molecularly Distinct

Alana Sorgini, Hugh Andrew Jinwook Kim, Peter YF Zeng, Mushfiq Hassan Shaikh, Neil Mundi, Farhad Ghasemi, Eric Di Gravio, Halema Khan, Danielle MacNeil, Mohammed Imran Khan, Adrian Mendez, John Yoo, Kevin Fung, Pencilla Lang, David A. Palma, Joe S. Mymryk, John W. Barrett, Krupal B. Patel, Paul C. Boutros and Anthony C. Nichols

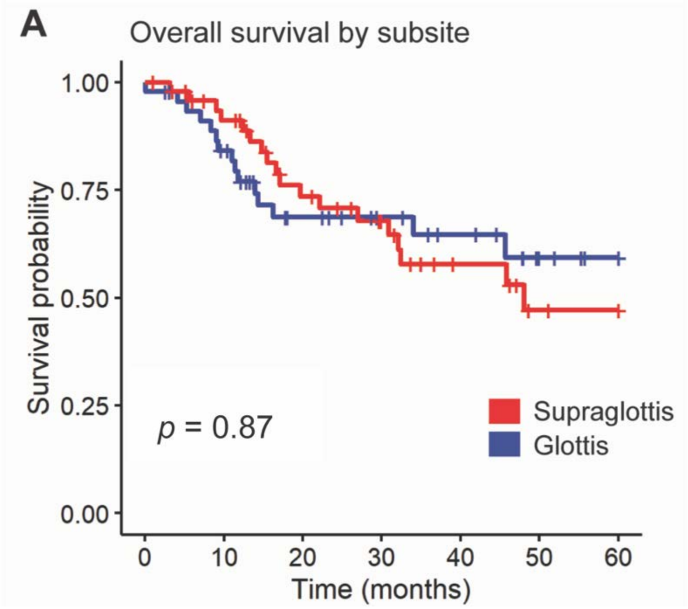

Figure S1. Kaplan-Meier curves for overall survival between supraglottic and glottic LSCC. A log-rank test was used to compare the Kaplan-Meier curves between tumors of the glottis and supraglottis.

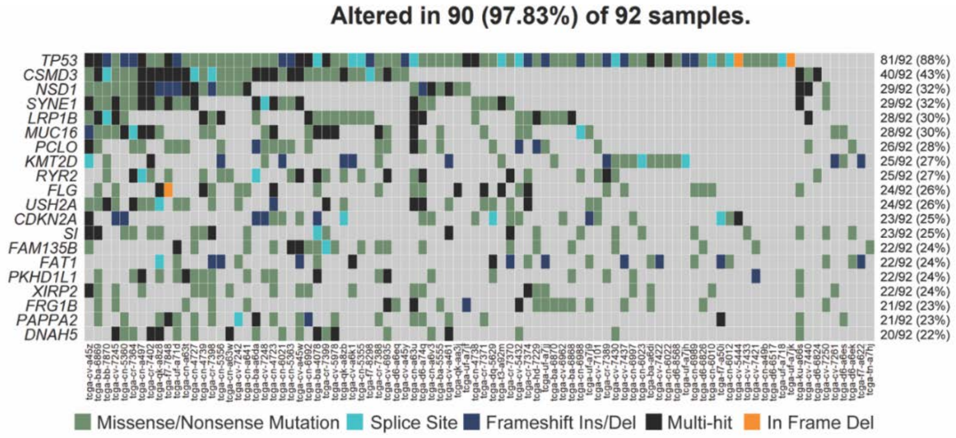

Figure S2. Top 20 genes by SNV frequency in the combined TCGA LSCC cohort. TCGA sample IDs, absolute number and frequency for each mutation are included.

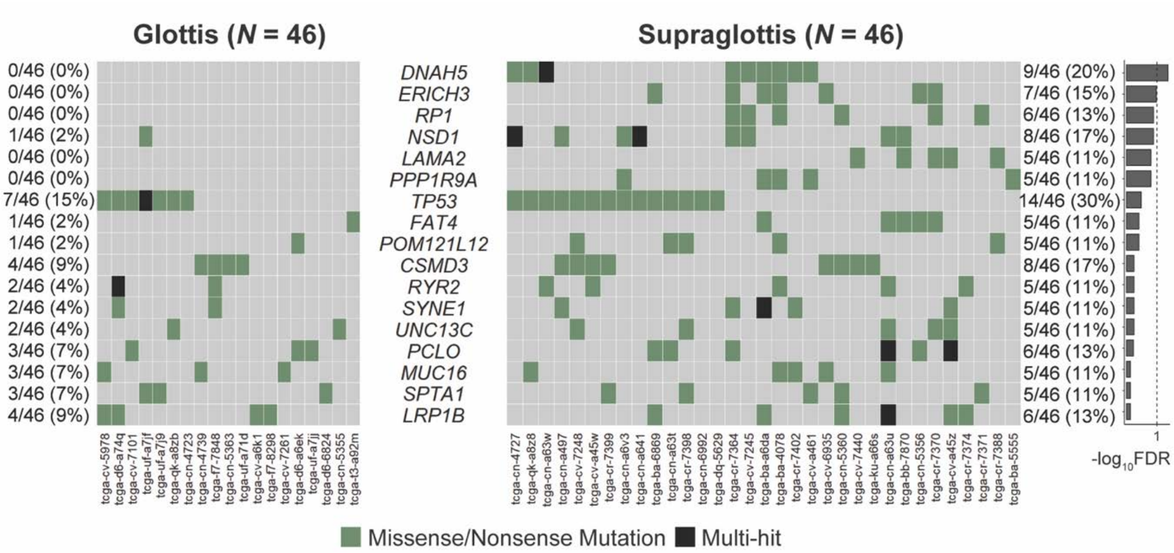

Figure S3. Genes with differing SNV mutation frequencies between supraglottic and glottic LSCC by G > T mutation load. FDR = false discovery rate.

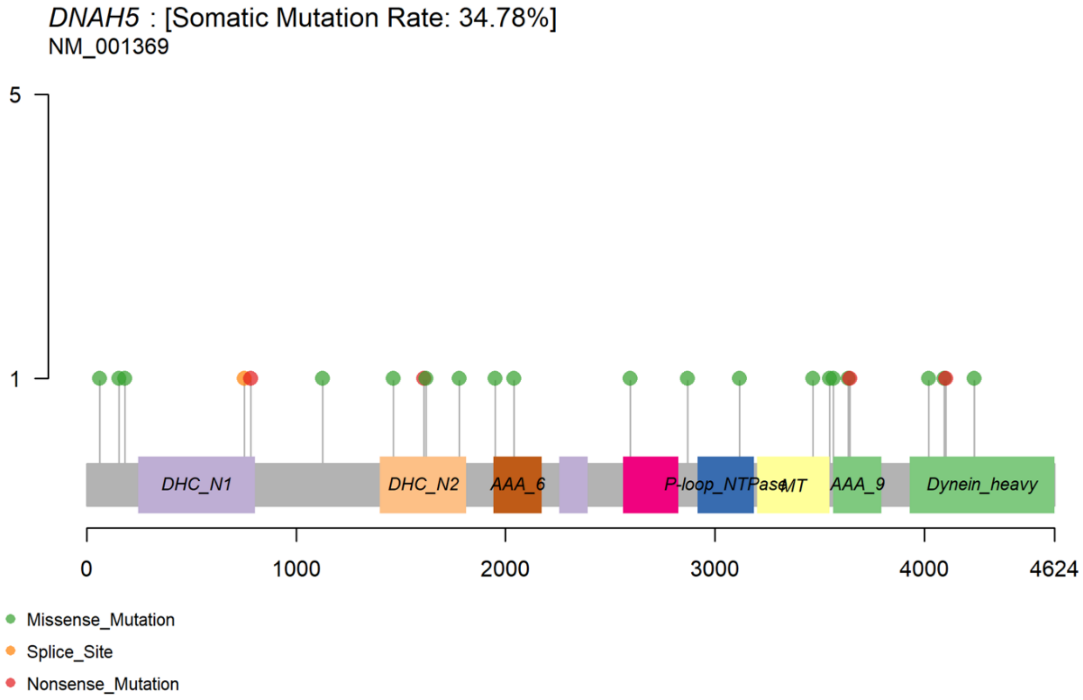

Figure S4. Lollipop plot of SNVs in *DNAH5*.

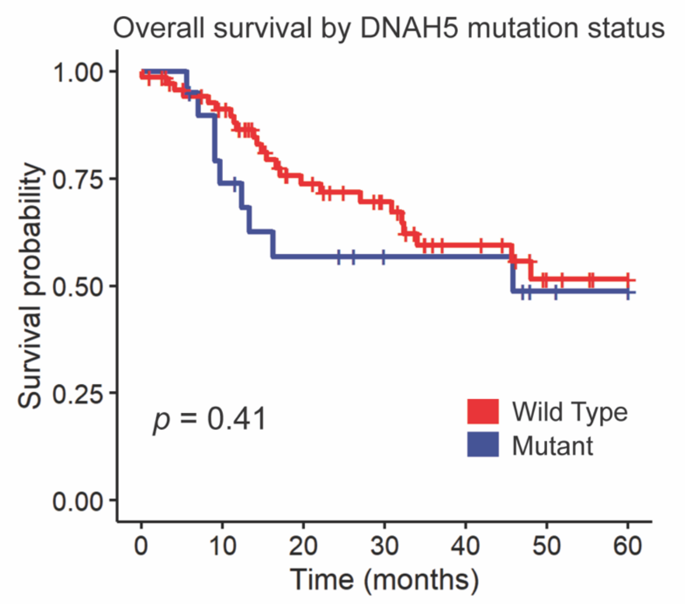

Figure S5. Kaplan-Meier curves for overall survival by *DNAH5* SNV mutation status. A log-rank test was used to compare the Kaplan-Meier curves between tumors with wild-type and mutant alleles.

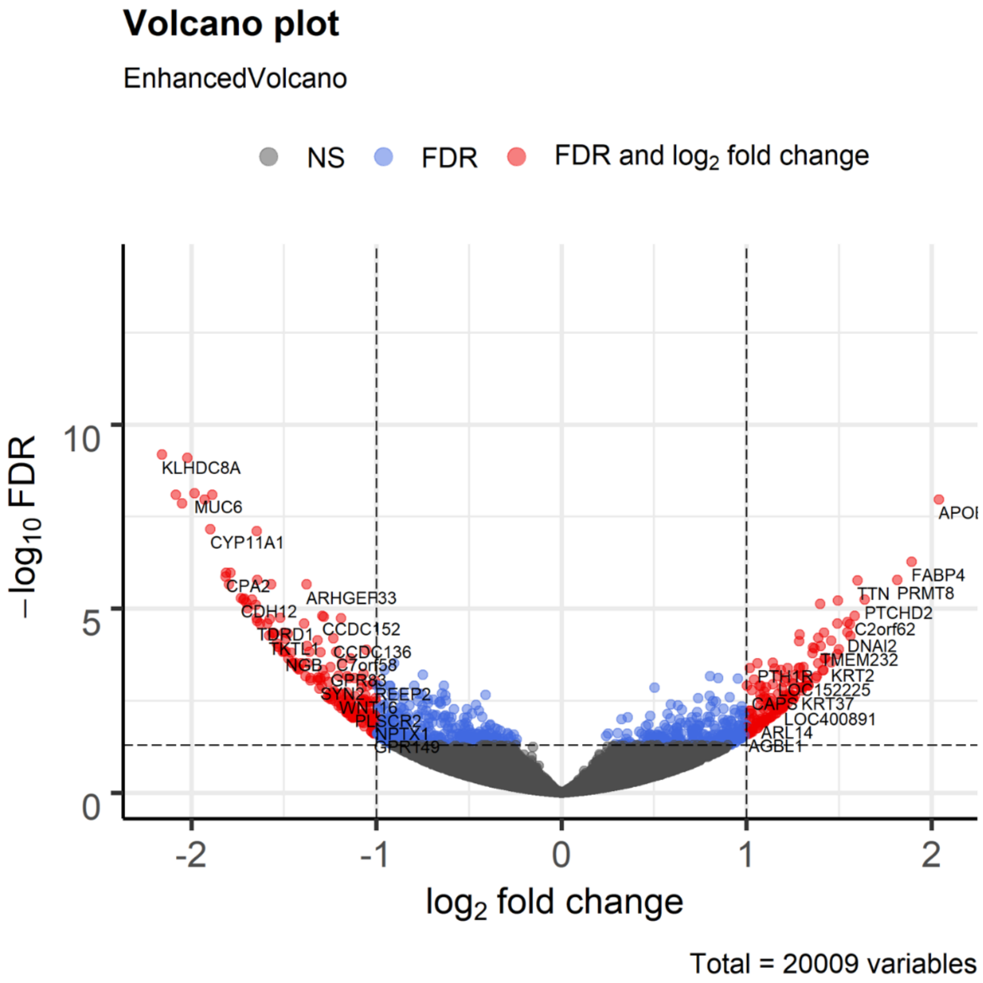

Figure S6. Volcano plot for mRNA abundance between glottic and supraglottic LSCC. 282 genes were found to have significant FDR-corrected significance values and greater than two-fold

change in transcript levels between glottic and supraglottic tumors. Genes with positive log2 fold change are more abundant in glottis, and vice versa.

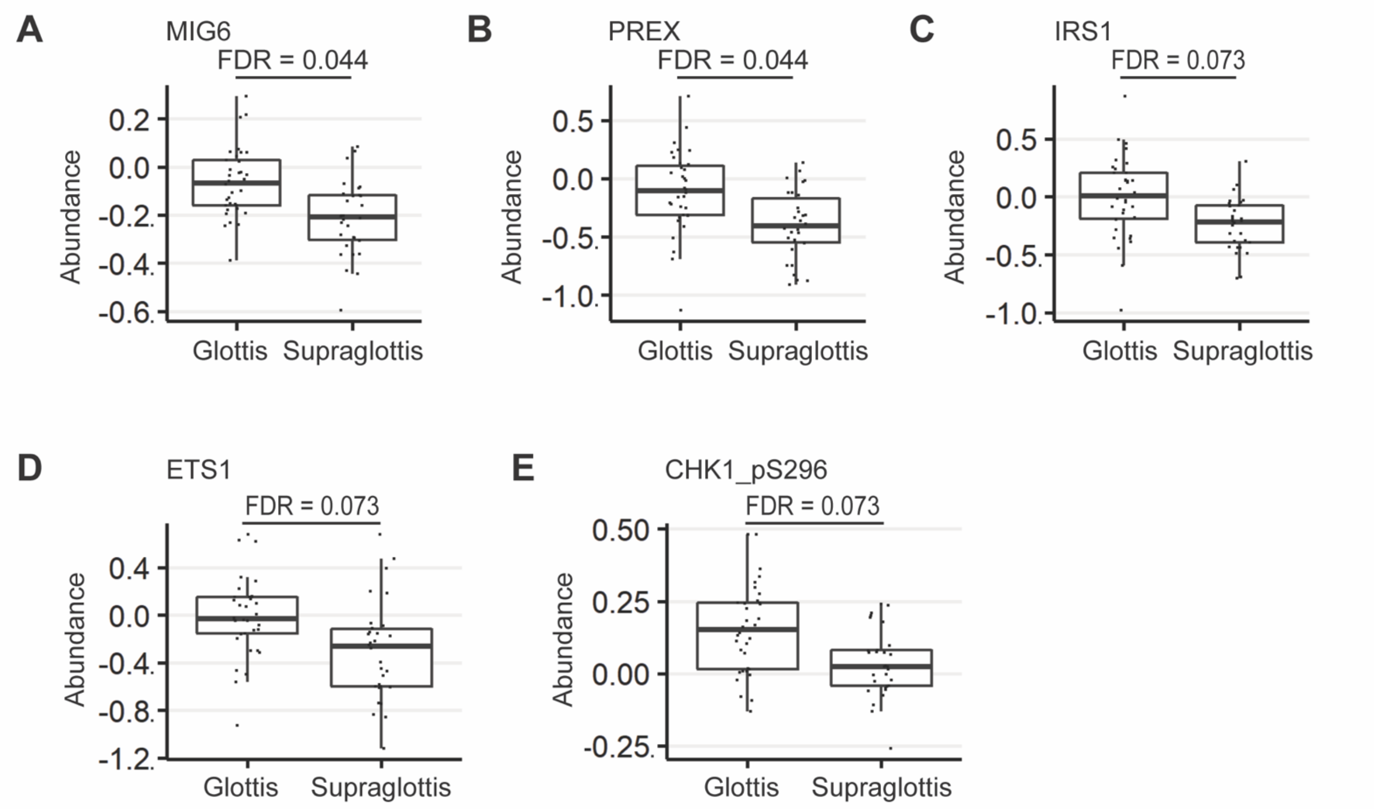

Figure S7. Differentially abundant proteins between supraglottic and glottic LSCC. Mann-Whitney U test was used to compare median protein abundance levels, and FDR correction was done using Benjamini-Hochberg method.

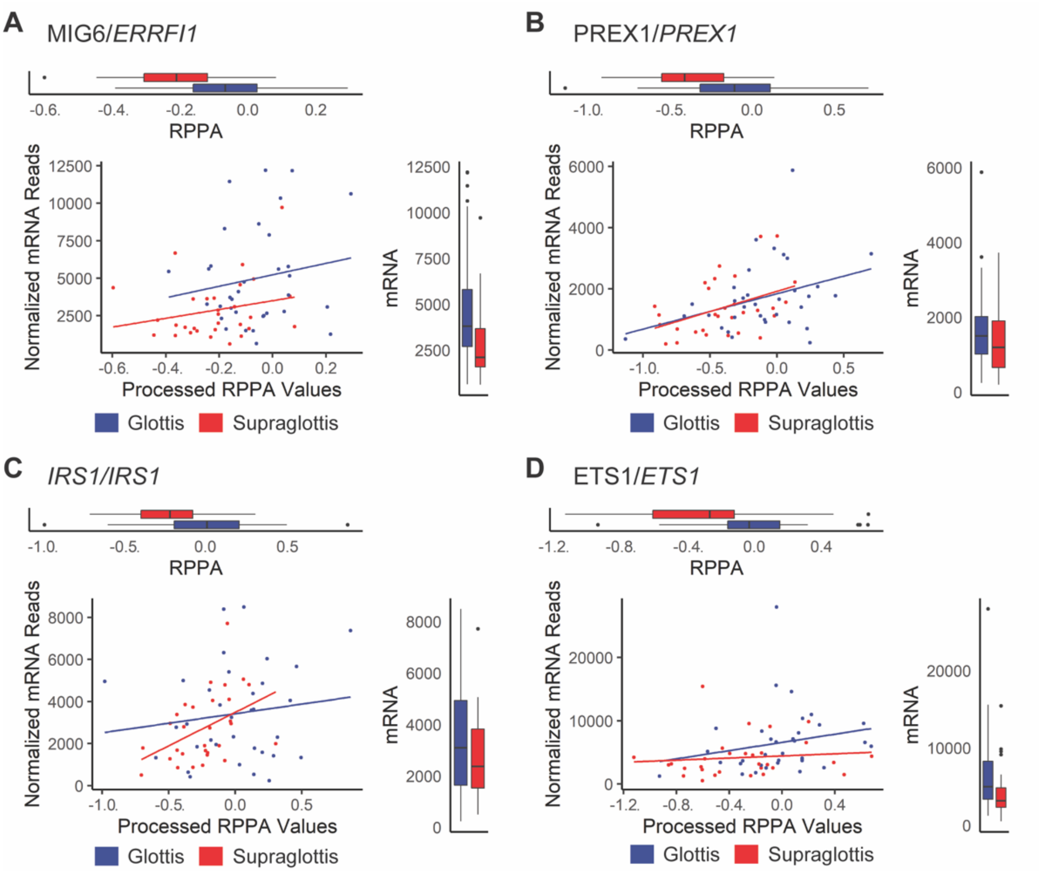

Figure S8. Four most differentially abundant proteins between supraglottic and glottic LSCC correlated with mRNA levels. Significance determined by FDR <0.1 and positive Spearman's correlation. (**A**) ERRFI1 (ϱ = 0.27, FDR = 0.046), (**B**) PREX1 (ϱ = 0.41, FDR = 0.0028), (**C**) IRS1 (ϱ = 0.26, FDR = 0.046), (**D**) ETS1 (ϱ = 0.40, FDR = 0.0028).

Table S1. Multivariate analysis of overall survival. Backward selection was used to attain the multivariate model. No multivariate model could be created in this way for progression-free survival.

**Variables**		**HPV-negative**			
		**Univariate**		**Multivariate**	
		**HR (95% CI)**	***p* Value**	**HR (95% CI)**	***p* Value**
**Age**	Mean	1.00 (0.96–1.04)	0.96		
**Sex**	Male vs. Female	0.24 (0.12–0.50)	$<10^{-3}$	0.17 (0.069–0.43)	**$<10^{-3}$**
**Smoking history**	Heavy vs. Light/None	0.30 (0.11–0.85)	0.023	0.19 (0.063–0.59)	**0.0039**
**T-category**	T4 vs. T2-T3	0.81 (0.37–1.8)	0.61		
**N-category**	N1-N3 vs. N0	3.2 (1.3–8.4)	0.016		
**Overall stage**	IV vs. I-III	0.86 (0.37–2.0)	0.72		
**Adjuvant radiotherapy**	Yes vs. No	0.69 (0.29–1.6)	0.40		
**Subsite**	Supraglottis vs. Glottis	1.2 (0.59–2.3)	0.66	2.7 (1.1–6.5)	**0.024**
**Cartilage invasion**	Positive vs. Negative	0.67 (0.31–1.5)	0.31		
**NSD1 mutation status**	Mutant vs. Wild-type	0.34 (0.14–0.82)	0.016	0.34 (0.12–0.95)	**0.039**

Table S2. List of genes with significantly different mRNA abundance or SNV frequencies between glottic and supraglottic LSCC. Significance cutoffs were <0.01 and absolute fold change >2 for mRNA and FDR<0.1 SNV comparisons.

**Gene**	**Log2 Fold Change or Odds Ratio**	***p* Value**	**FDR**	**Mutation Difference**
APOBEC2	2.0	3.9×10^{-12}	1.1×10^{-8}	higher mRNA abundance in glottis
FABP4	1.9	3.0×10^{-10}	5.2×10^{-7}	higher mRNA abundance in glottis
PRMT8	1.8	1.4×10^{-9}	1.6×10^{-6}	higher mRNA abundance in glottis
TTN	1.6	1.5×10^{-9}	1.7×10^{-6}	higher mRNA abundance in glottis

PTCHD2	1.6	6.9×10^{-9}	5.6×10^{-6}	higher mRNA abundance in glottis
DHRS2	1.5	8.2×10^{-9}	5.9×10^{-6}	higher mRNA abundance in glottis
MYOM1	1.4	1.1×10^{-8}	7.3×10^{-6}	higher mRNA abundance in glottis
C2orf62	1.6	2.6×10^{-8}	1.5×10^{-5}	higher mRNA abundance in glottis
VWA3B	1.5	4.7×10^{-8}	2.3×10^{-5}	higher mRNA abundance in glottis
ZMYND10	1.5	5.5×10^{-8}	2.5×10^{-5}	higher mRNA abundance in glottis
KRT84	1.6	5.9×10^{-8}	2.5×10^{-5}	higher mRNA abundance in glottis
DNAI2	1.5	1.0×10^{-7}	4.3×10^{-5}	higher mRNA abundance in glottis
MURC	1.4	1.1×10^{-7}	4.4×10^{-5}	higher mRNA abundance in glottis
C8orf48	1.3	1.4×10^{-7}	4.9×10^{-5}	higher mRNA abundance in glottis
CDHR4	1.6	1.6×10^{-7}	5.5×10^{-5}	higher mRNA abundance in glottis
CDH15	1.4	1.8×10^{-7}	6.1×10^{-5}	higher mRNA abundance in glottis
RP1	1.5	2.3×10^{-7}	7.4×10^{-5}	higher mRNA abundance in glottis
STEAP4	1.3	2.4×10^{-7}	7.5×10^{-5}	higher mRNA abundance in glottis
TMEM232	1.4	3.4×10^{-7}	1.0×10^{-4}	higher mRNA abundance in glottis
CCDC65	1.4	4.0×10^{-7}	1.1×10^{-4}	higher mRNA abundance in glottis
CLVS1	1.4	4.2×10^{-7}	1.2×10^{-4}	higher mRNA abundance in glottis
SLN	1.5	4.6×10^{-7}	1.3×10^{-4}	higher mRNA abundance in glottis
LRRC50	1.4	6.6×10^{-7}	1.6×10^{-4}	higher mRNA abundance in glottis
CCDC108	1.5	7.0×10^{-7}	1.7×10^{-4}	higher mRNA abundance in glottis
CCK	1.4	9.1×10^{-7}	2.2×10^{-4}	higher mRNA abundance in glottis
CXorf64	1.4	9.7×10^{-7}	2.3×10^{-4}	higher mRNA abundance in glottis
KRT2	1.5	1.2×10^{-6}	2.8×10^{-4}	higher mRNA abundance in glottis
LRRC46	1.1	1.3×10^{-6}	3.0×10^{-4}	higher mRNA abundance in glottis
PTH1R	1.1	1.4×10^{-6}	3.0×10^{-4}	higher mRNA abundance in glottis
SERPINA9	1.4	1.4×10^{-6}	3.0×10^{-4}	higher mRNA abundance in glottis
C2orf40	1.3	1.9×10^{-6}	3.9×10^{-4}	higher mRNA abundance in glottis
CRB1	1.3	2.0×10^{-6}	4.0×10^{-4}	higher mRNA abundance in glottis
FRMPD1	1.2	2.1×10^{-6}	4.0×10^{-4}	higher mRNA abundance in glottis
**Gene**	**Log2 Fold Change or Odds Ratio**	***p* Value**	**FDR**	**Mutation Difference**
SLC22A3	1.3	2.2×10^{-6}	4.1×10^{-4}	higher mRNA abundance in glottis
TUSC1	1.0	2.2×10^{-6}	4.1×10^{-4}	higher mRNA abundance in glottis
C6orf97	1.2	2.3×10^{-6}	4.2×10^{-4}	higher mRNA abundance in glottis
VWA3A	1.4	2.6×10^{-6}	4.6×10^{-4}	higher mRNA abundance in glottis
CCDC17	1.2	2.6×10^{-6}	4.6×10^{-4}	higher mRNA abundance in glottis
DNAH12	1.4	2.7×10^{-6}	4.7×10^{-4}	higher mRNA abundance in glottis
MYL4	1.3	3.6×10^{-6}	6.1×10^{-4}	higher mRNA abundance in glottis
ROPN1L	1.3	3.8×10^{-6}	6.4×10^{-4}	higher mRNA abundance in glottis
LOC152225	1.2	4.2×10^{-6}	6.8×10^{-4}	higher mRNA abundance in glottis
RSPH1	1.3	4.2×10^{-6}	6.8×10^{-4}	higher mRNA abundance in glottis
MYOT	1.4	4.3×10^{-6}	6.8×10^{-4}	higher mRNA abundance in glottis
DIO3	1.2	4.9×10^{-6}	7.6×10^{-4}	higher mRNA abundance in glottis
PRB2	1.4	5.1×10^{-6}	7.6×10^{-4}	higher mRNA abundance in glottis
LCE3E	1.3	5.3×10^{-6}	7.7×10^{-4}	higher mRNA abundance in glottis
LRRC6	1.0	5.8×10^{-6}	8.3×10^{-4}	higher mRNA abundance in glottis
WDR63	1.2	7.1×10^{-6}	9.7×10^{-4}	higher mRNA abundance in glottis
KRT10	1.2	7.7×10^{-6}	1.0×10^{-3}	higher mRNA abundance in glottis
FAM166B	1.1	8.3×10^{-6}	1.1×10^{-3}	higher mRNA abundance in glottis
MYH3	1.3	8.3×10^{-6}	1.1×10^{-3}	higher mRNA abundance in glottis
DNAI1	1.3	9.0×10^{-6}	1.2×10^{-3}	higher mRNA abundance in glottis
C11orf66	1.0	9.4×10^{-6}	1.2×10^{-3}	higher mRNA abundance in glottis
MYPN	1.3	9.5×10^{-6}	1.2×10^{-3}	higher mRNA abundance in glottis
CASQ2	1.3	9.8×10^{-6}	1.2×10^{-3}	higher mRNA abundance in glottis
FCAMR	1.3	9.9×10^{-6}	1.2×10^{-3}	higher mRNA abundance in glottis

MT2A	1.1	1.0×10^{-5}	1.2×10^{-3}	higher mRNA abundance in glottis
RSPH9	1.1	1.0×10^{-5}	1.2×10^{-3}	higher mRNA abundance in glottis
MME	1.1	1.1×10^{-5}	1.3×10^{-3}	higher mRNA abundance in glottis
TNNC2	1.3	1.1×10^{-5}	1.3×10^{-3}	higher mRNA abundance in glottis
TMEM190	1.3	1.2×10^{-5}	1.4×10^{-3}	higher mRNA abundance in glottis
HMP19	1.2	1.2×10^{-5}	1.4×10^{-3}	higher mRNA abundance in glottis
CAPS	1.0	1.4×10^{-5}	1.6×10^{-3}	higher mRNA abundance in glottis
KRT37	1.3	1.5×10^{-5}	1.7×10^{-3}	higher mRNA abundance in glottis
HSPA7	1.1	1.6×10^{-5}	1.8×10^{-3}	higher mRNA abundance in glottis
S100A7	1.2	1.8×10^{-5}	2.0×10^{-3}	higher mRNA abundance in glottis
SPEF1	1.2	1.9×10^{-5}	2.1×10^{-3}	higher mRNA abundance in glottis
HSPA6	1.1	2.1×10^{-5}	2.2×10^{-3}	higher mRNA abundance in glottis
ATP1A2	1.2	2.1×10^{-5}	2.2×10^{-3}	higher mRNA abundance in glottis
**Gene**	**Log2 Fold Change or Odds Ratio**	***p* Value**	**FDR**	**Mutation Difference**
LCE3D	1.2	2.2×10^{-5}	2.2×10^{-3}	higher mRNA abundance in glottis
MYH1	1.2	2.3×10^{-5}	2.3×10^{-3}	higher mRNA abundance in glottis
CHGB	1.3	2.4×10^{-5}	2.3×10^{-3}	higher mRNA abundance in glottis
CHRNA1	1.3	2.4×10^{-5}	2.4×10^{-3}	higher mRNA abundance in glottis
C5orf49	1.2	2.5×10^{-5}	2.4×10^{-3}	higher mRNA abundance in glottis
NOG	1.2	2.6×10^{-5}	2.5×10^{-3}	higher mRNA abundance in glottis
KCNJ13	1.1	2.7×10^{-5}	2.5×10^{-3}	higher mRNA abundance in glottis
UTS2	1.1	2.9×10^{-5}	2.7×10^{-3}	higher mRNA abundance in glottis
FAM154B	1.1	3.0×10^{-5}	2.8×10^{-3}	higher mRNA abundance in glottis
CADM3	1.1	3.1×10^{-5}	2.8×10^{-3}	higher mRNA abundance in glottis
SNTN	1.3	3.2×10^{-5}	2.9×10^{-3}	higher mRNA abundance in glottis
SPINLW1	1.3	3.3×10^{-5}	2.9×10^{-3}	higher mRNA abundance in glottis
PPP1R3A	1.2	3.7×10^{-5}	3.2×10^{-3}	higher mRNA abundance in glottis
KRT34	1.1	3.9×10^{-5}	3.3×10^{-3}	higher mRNA abundance in glottis
TSPAN19	1.2	3.9×10^{-5}	3.3×10^{-3}	higher mRNA abundance in glottis
LMOD3	1.2	4.6×10^{-5}	3.6×10^{-3}	higher mRNA abundance in glottis
S100A8	1.1	4.7×10^{-5}	3.6×10^{-3}	higher mRNA abundance in glottis
LOC400891	1.2	5.6×10^{-5}	4.2×10^{-3}	higher mRNA abundance in glottis
TEKT1	1.1	5.6×10^{-5}	4.2×10^{-3}	higher mRNA abundance in glottis
SPRR2G	1.2	5.7×10^{-5}	4.2×10^{-3}	higher mRNA abundance in glottis
PRB1	1.2	5.7×10^{-5}	4.3×10^{-3}	higher mRNA abundance in glottis
CST6	1.1	5.8×10^{-5}	4.3×10^{-3}	higher mRNA abundance in glottis
C10orf71	1.2	6.3×10^{-5}	4.5×10^{-3}	higher mRNA abundance in glottis
FRMPD2	1.2	6.5×10^{-5}	4.6×10^{-3}	higher mRNA abundance in glottis
TRHDE	1.2	6.5×10^{-5}	4.6×10^{-3}	higher mRNA abundance in glottis
GAS2L2	1.1	6.7×10^{-5}	4.7×10^{-3}	higher mRNA abundance in glottis
PZP	1.2	6.8×10^{-5}	4.7×10^{-3}	higher mRNA abundance in glottis
MYBPC2	1.2	7.3×10^{-5}	5.0×10^{-3}	higher mRNA abundance in glottis
SLC25A2	1.1	7.8×10^{-5}	5.2×10^{-3}	higher mRNA abundance in glottis
NME5	1.1	7.8×10^{-5}	5.2×10^{-3}	higher mRNA abundance in glottis
MORN5	1.2	7.9×10^{-5}	5.2×10^{-3}	higher mRNA abundance in glottis
ROS1	1.2	8.2×10^{-5}	5.4×10^{-3}	higher mRNA abundance in glottis
C6orf58	1.2	8.4×10^{-5}	5.4×10^{-3}	higher mRNA abundance in glottis
CR2	1.1	8.6×10^{-5}	5.5×10^{-3}	higher mRNA abundance in glottis
CHRNG	1.2	8.6×10^{-5}	5.6×10^{-3}	higher mRNA abundance in glottis
ANKRD1	1.2	8.7×10^{-5}	5.6×10^{-3}	higher mRNA abundance in glottis
WDR65	1.0	9.2×10^{-5}	5.8×10^{-3}	higher mRNA abundance in glottis
**Gene**	**Log2 Fold Change or Odds Ratio**	***p* Value**	**FDR**	**Mutation Difference**
STAC3	1.0	9.3×10^{-5}	5.8×10^{-3}	higher mRNA abundance in glottis
TNNI1	1.1	1.1×10^{-4}	6.6×10^{-3}	higher mRNA abundance in glottis

MYLPF	1.2	1.1×10^{-4}	6.8×10^{-3}	higher mRNA abundance in glottis
SLPI	1.0	1.2×10^{-4}	6.9×10^{-3}	higher mRNA abundance in glottis
C14orf34	1.0	1.2×10^{-4}	7.0×10^{-3}	higher mRNA abundance in glottis
PRKAG3	1.2	1.2×10^{-4}	7.0×10^{-3}	higher mRNA abundance in glottis
C13orf30	1.1	1.3×10^{-4}	7.6×10^{-3}	higher mRNA abundance in glottis
CACNG6	1.1	1.6×10^{-4}	8.6×10^{-3}	higher mRNA abundance in glottis
CACNG1	1.1	1.6×10^{-4}	8.7×10^{-3}	higher mRNA abundance in glottis
ACTC1	1.1	1.8×10^{-4}	9.1×10^{-3}	higher mRNA abundance in glottis
DCDC2B	1.1	1.8×10^{-4}	9.3×10^{-3}	higher mRNA abundance in glottis
SPATA4	1.1	1.9×10^{-4}	9.3×10^{-3}	higher mRNA abundance in glottis
NCAM1	1.0	1.9×10^{-4}	9.3×10^{-3}	higher mRNA abundance in glottis
ARL14	1.1	2.0×10^{-4}	9.8×10^{-3}	higher mRNA abundance in glottis
BEST3	1.1	2.0×10^{-4}	9.9×10^{-3}	higher mRNA abundance in glottis
KLHDC8A	−2.2	3.4×10^{-14}	6.3×10^{-10}	higher mRNA abundance in supraglottis
CHRM5	−2.0	8.4×10^{-14}	7.9×10^{-10}	higher mRNA abundance in supraglottis
MUC6	−2.0	1.2×10^{-12}	7.2×10^{-9}	higher mRNA abundance in supraglottis
HRASLS5	−2.1	1.7×10^{-12}	8.0×10^{-9}	higher mRNA abundance in supraglottis
SLC4A8	−1.9	2.1×10^{-12}	8.0×10^{-9}	higher mRNA abundance in supraglottis
SLC13A4	−1.9	3.8×10^{-12}	1.1×10^{-8}	higher mRNA abundance in supraglottis
CALHM3	−2.1	5.7×10^{-12}	1.3×10^{-8}	higher mRNA abundance in supraglottis
CYP11A1	−1.9	3.2×10^{-11}	6.7×10^{-8}	higher mRNA abundance in supraglottis
PIPOX	−1.6	4.1×10^{-11}	7.7×10^{-8}	higher mRNA abundance in supraglottis
CPA2	−1.8	6.9×10^{-10}	1.0×10^{-6}	higher mRNA abundance in supraglottis
SVOP	−1.8	7.1×10^{-10}	1.0×10^{-6}	higher mRNA abundance in supraglottis
CLCNKA	−1.8	9.8×10^{-10}	1.3×10^{-6}	higher mRNA abundance in supraglottis
CTCFL	−1.6	1.4×10^{-9}	1.6×10^{-6}	higher mRNA abundance in supraglottis
ARHGEF33	−1.4	2.2×10^{-9}	2.1×10^{-6}	higher mRNA abundance in supraglottis
TMEM151B	−1.6	2.2×10^{-9}	2.1×10^{-6}	higher mRNA abundance in supraglottis
VIL1	−1.8	2.3×10^{-9}	2.1×10^{-6}	higher mRNA abundance in supraglottis
CDH12	−1.7	5.6×10^{-9}	5.0×10^{-6}	higher mRNA abundance in supraglottis
KCNJ4	−1.7	6.2×10^{-9}	5.3×10^{-6}	higher mRNA abundance in supraglottis
FTCD	−1.7	7.2×10^{-9}	5.6×10^{-6}	higher mRNA abundance in supraglottis
PNMA5	−1.7	7.4×10^{-9}	5.6×10^{-6}	higher mRNA abundance in supraglottis
UROC1	−1.7	9.4×10^{-9}	6.6×10^{-6}	higher mRNA abundance in supraglottis
THSD7B	−1.7	1.2×10^{-8}	7.8×10^{-6}	higher mRNA abundance in supraglottis
**Gene**	**Log2 Fold Change or Odds Ratio**	***p* Value**	**FDR**	**Mutation Difference**
TDRD12	−1.7	1.5×10^{-8}	9.6×10^{-6}	higher mRNA abundance in supraglottis
CCDC152	−1.3	2.6×10^{-8}	1.5×10^{-5}	higher mRNA abundance in supraglottis
LOC387646	−1.3	2.9×10^{-8}	1.6×10^{-5}	higher mRNA abundance in supraglottis
SERPINI1	−1.5	3.2×10^{-8}	1.8×10^{-5}	higher mRNA abundance in supraglottis
RCOR2	−1.2	3.4×10^{-8}	1.8×10^{-5}	higher mRNA abundance in supraglottis
DCAF12L2	−1.6	3.4×10^{-8}	1.8×10^{-5}	higher mRNA abundance in supraglottis
DLGAP1	−1.6	3.8×10^{-8}	1.9×10^{-5}	higher mRNA abundance in supraglottis
TDRD1	−1.6	4.3×10^{-8}	2.1×10^{-5}	higher mRNA abundance in supraglottis
LOC84856	−1.4	5.5×10^{-8}	2.5×10^{-5}	higher mRNA abundance in supraglottis
WFIKKN2	−1.6	5.6×10^{-8}	2.5×10^{-5}	higher mRNA abundance in supraglottis
NAP1L6	−1.6	5.7×10^{-8}	2.5×10^{-5}	higher mRNA abundance in supraglottis
NEUROD2	−1.6	1.1×10^{-7}	4.4×10^{-5}	higher mRNA abundance in supraglottis
NKX2	−1.5	1.1×10^{-7}	4.4×10^{-5}	higher mRNA abundance in supraglottis
PENK	−1.6	1.2×10^{-7}	4.6×10^{-5}	higher mRNA abundance in supraglottis
CYP26C1	−1.6	1.3×10^{-7}	4.7×10^{-5}	higher mRNA abundance in supraglottis
ABCG5	−1.5	1.3×10^{-7}	4.7×10^{-5}	higher mRNA abundance in supraglottis
TKTL1	−1.6	1.5×10^{-7}	5.3×10^{-5}	higher mRNA abundance in supraglottis
CCDC136	−1.2	1.9×10^{-7}	6.4×10^{-5}	higher mRNA abundance in supraglottis

ANO3	−1.5	1.9×10^{-7}	6.4×10^{-5}	higher mRNA abundance in supraglottis
MYB	−1.3	2.2×10^{-7}	7.2×10^{-5}	higher mRNA abundance in supraglottis
PCSK2	−1.5	2.8×10^{-7}	8.7×10^{-5}	higher mRNA abundance in supraglottis
C10orf105	−1.4	3.5×10^{-7}	1.0×10^{-4}	higher mRNA abundance in supraglottis
FGF21	−1.5	3.7×10^{-7}	1.1×10^{-4}	higher mRNA abundance in supraglottis
CTRB2	−1.5	3.8×10^{-7}	1.1×10^{-4}	higher mRNA abundance in supraglottis
ZNF542	−1.1	4.8×10^{-7}	1.3×10^{-4}	higher mRNA abundance in supraglottis
NGB	−1.5	5.3×10^{-7}	1.4×10^{-4}	higher mRNA abundance in supraglottis
F2	−1.5	5.5×10^{-7}	1.4×10^{-4}	higher mRNA abundance in supraglottis
GRIK4	−1.4	5.6×10^{-7}	1.4×10^{-4}	higher mRNA abundance in supraglottis
C7orf58	−1.2	5.7×10^{-7}	1.4×10^{-4}	higher mRNA abundance in supraglottis
TUBA3E	−1.5	5.7×10^{-7}	1.4×10^{-4}	higher mRNA abundance in supraglottis
LMO3	−1.3	6.0×10^{-7}	1.5×10^{-4}	higher mRNA abundance in supraglottis
HAPLN2	−1.5	6.3×10^{-7}	1.6×10^{-4}	higher mRNA abundance in supraglottis
FGFBP2	−1.5	9.3×10^{-7}	2.2×10^{-4}	higher mRNA abundance in supraglottis
CABP1	−1.1	9.8×10^{-7}	2.3×10^{-4}	higher mRNA abundance in supraglottis
LCT	−1.4	1.3×10^{-6}	3.0×10^{-4}	higher mRNA abundance in supraglottis
KRT71	−1.4	1.4×10^{-6}	3.0×10^{-4}	higher mRNA abundance in supraglottis
KRT20	−1.5	1.4×10^{-6}	3.0×10^{-4}	higher mRNA abundance in supraglottis
**Gene**	**Log2 Fold Change or Odds Ratio**	***p* Value**	**FDR**	**Mutation Difference**
LOC283999	−1.5	1.5×10^{-6}	3.0×10^{-4}	higher mRNA abundance in supraglottis
CABP7	−1.2	1.5×10^{-6}	3.0×10^{-4}	higher mRNA abundance in supraglottis
PON3	−1.4	1.5×10^{-6}	3.0×10^{-4}	higher mRNA abundance in supraglottis
GPR83	−1.2	1.9×10^{-6}	3.8×10^{-4}	higher mRNA abundance in supraglottis
CPA1	−1.4	2.0×10^{-6}	4.0×10^{-4}	higher mRNA abundance in supraglottis
ABCG8	−1.4	2.2×10^{-6}	4.1×10^{-4}	higher mRNA abundance in supraglottis
LY6G6F	−1.4	2.4×10^{-6}	4.4×10^{-4}	higher mRNA abundance in supraglottis
SRMS	−1.3	2.5×10^{-6}	4.5×10^{-4}	higher mRNA abundance in supraglottis
IQCG	−1.1	3.4×10^{-6}	5.8×10^{-4}	higher mRNA abundance in supraglottis
KCNN1	−1.2	3.9×10^{-6}	6.6×10^{-4}	higher mRNA abundance in supraglottis
PLCH1	−1.2	4.2×10^{-6}	6.8×10^{-4}	higher mRNA abundance in supraglottis
TMPRSS15	−1.4	4.3×10^{-6}	6.8×10^{-4}	higher mRNA abundance in supraglottis
psiTPTE22	−1.3	4.3×10^{-6}	6.8×10^{-4}	higher mRNA abundance in supraglottis
TRPM5	−1.3	4.8×10^{-6}	7.4×10^{-4}	higher mRNA abundance in supraglottis
OR1F1	−1.3	4.9×10^{-6}	7.5×10^{-4}	higher mRNA abundance in supraglottis
TUBAL3	−1.1	5.0×10^{-6}	7.6×10^{-4}	higher mRNA abundance in supraglottis
OLFM4	−1.4	5.0×10^{-6}	7.6×10^{-4}	higher mRNA abundance in supraglottis
PCSK4	−1.1	5.2×10^{-6}	7.7×10^{-4}	higher mRNA abundance in supraglottis
SYN2	−1.3	6.0×10^{-6}	8.6×10^{-4}	higher mRNA abundance in supraglottis
BSND	−1.3	6.1×10^{-6}	8.7×10^{-4}	higher mRNA abundance in supraglottis
DEPDC6	−1.1	6.2×10^{-6}	8.8×10^{-4}	higher mRNA abundance in supraglottis
SLC30A8	−1.4	6.4×10^{-6}	9.0×10^{-4}	higher mRNA abundance in supraglottis
REEP2	−1.0	6.5×10^{-6}	9.1×10^{-4}	higher mRNA abundance in supraglottis
LOC284578	−1.3	7.0×10^{-6}	9.6×10^{-4}	higher mRNA abundance in supraglottis
WNT10B	−1.0	8.6×10^{-6}	1.1×10^{-3}	higher mRNA abundance in supraglottis
GCK	−1.1	9.4×10^{-6}	1.2×10^{-3}	higher mRNA abundance in supraglottis
LIN28B	−1.3	1.0×10^{-5}	1.2×10^{-3}	higher mRNA abundance in supraglottis
INHBE	−1.1	1.2×10^{-5}	1.4×10^{-3}	higher mRNA abundance in supraglottis
SHH	−1.3	1.3×10^{-5}	1.5×10^{-3}	higher mRNA abundance in supraglottis
ADCY8	−1.2	1.5×10^{-5}	1.7×10^{-3}	higher mRNA abundance in supraglottis
SLC1A2	−1.2	1.7×10^{-5}	1.9×10^{-3}	higher mRNA abundance in supraglottis
C15orf55	−1.3	1.7×10^{-5}	1.9×10^{-3}	higher mRNA abundance in supraglottis
WNT8B	−1.2	1.8×10^{-5}	1.9×10^{-3}	higher mRNA abundance in supraglottis
ZNF883	−1.2	1.9×10^{-5}	2.0×10^{-3}	higher mRNA abundance in supraglottis

WNT16	−1.2	2.0×10^{-5}	2.1×10^{-3}	higher mRNA abundance in supraglottis
MT3	−1.3	2.1×10^{-5}	2.2×10^{-3}	higher mRNA abundance in supraglottis
FA2H	−1.1	2.3×10^{-5}	2.3×10^{-3}	higher mRNA abundance in supraglottis
**Gene**	**Log2 Fold Change or Odds Ratio**	***p* Value**	**FDR**	**Mutation Difference**
ANKRD34B	−1.2	2.3×10^{-5}	2.3×10^{-3}	higher mRNA abundance in supraglottis
SLC8A2	−1.1	2.5×10^{-5}	2.4×10^{-3}	higher mRNA abundance in supraglottis
LIM2	−1.3	2.6×10^{-5}	2.5×10^{-3}	higher mRNA abundance in supraglottis
MUC17	−1.3	2.7×10^{-5}	2.5×10^{-3}	higher mRNA abundance in supraglottis
ZYG11A	−1.2	2.7×10^{-5}	2.6×10^{-3}	higher mRNA abundance in supraglottis
RPS2P32	−1.0	2.9×10^{-5}	2.7×10^{-3}	higher mRNA abundance in supraglottis
DBX2	−1.2	3.1×10^{-5}	2.8×10^{-3}	higher mRNA abundance in supraglottis
TMEM35	−1.1	3.2×10^{-5}	2.8×10^{-3}	higher mRNA abundance in supraglottis
ZNF280B	−1.1	3.2×10^{-5}	2.9×10^{-3}	higher mRNA abundance in supraglottis
CPSF4L	−1.2	3.3×10^{-5}	2.9×10^{-3}	higher mRNA abundance in supraglottis
FBXL13	−1.1	3.4×10^{-5}	2.9×10^{-3}	higher mRNA abundance in supraglottis
TULP2	−1.2	3.5×10^{-5}	3.0×10^{-3}	higher mRNA abundance in supraglottis
TSPYL5	−1.1	3.6×10^{-5}	3.1×10^{-3}	higher mRNA abundance in supraglottis
POU4F1	−1.2	3.7×10^{-5}	3.2×10^{-3}	higher mRNA abundance in supraglottis
GPAT2	−1.1	3.8×10^{-5}	3.2×10^{-3}	higher mRNA abundance in supraglottis
DNAJC22	−1.0	4.0×10^{-5}	3.3×10^{-3}	higher mRNA abundance in supraglottis
MLC1	−1.0	4.0×10^{-5}	3.3×10^{-3}	higher mRNA abundance in supraglottis
NCRNA00202	−1.2	4.2×10^{-5}	3.4×10^{-3}	higher mRNA abundance in supraglottis
MYH7B	−1.1	4.2×10^{-5}	3.4×10^{-3}	higher mRNA abundance in supraglottis
MOXD1	−1.1	4.2×10^{-5}	3.4×10^{-3}	higher mRNA abundance in supraglottis
C6orf221	−1.2	4.3×10^{-5}	3.5×10^{-3}	higher mRNA abundance in supraglottis
DPP4	−1.1	4.5×10^{-5}	3.6×10^{-3}	higher mRNA abundance in supraglottis
C2orf65	−1.2	4.6×10^{-5}	3.6×10^{-3}	higher mRNA abundance in supraglottis
COL21A1	−1.2	4.8×10^{-5}	3.8×10^{-3}	higher mRNA abundance in supraglottis
GIP	−1.2	5.0×10^{-5}	3.8×10^{-3}	higher mRNA abundance in supraglottis
BEST2	−1.2	5.8×10^{-5}	4.3×10^{-3}	higher mRNA abundance in supraglottis
HPN	−1.2	5.8×10^{-5}	4.3×10^{-3}	higher mRNA abundance in supraglottis
ZIC4	−1.2	5.9×10^{-5}	4.3×10^{-3}	higher mRNA abundance in supraglottis
TCEB3B	−1.2	6.1×10^{-5}	4.4×10^{-3}	higher mRNA abundance in supraglottis
GRB14	−1.1	6.4×10^{-5}	4.6×10^{-3}	higher mRNA abundance in supraglottis
PLSCR2	−1.1	6.7×10^{-5}	4.7×10^{-3}	higher mRNA abundance in supraglottis
PCP4	−1.1	6.9×10^{-5}	4.8×10^{-3}	higher mRNA abundance in supraglottis
HBE1	−1.1	7.0×10^{-5}	4.8×10^{-3}	higher mRNA abundance in supraglottis
CDH18	−1.2	7.4×10^{-5}	5.1×10^{-3}	higher mRNA abundance in supraglottis
FGF12	−1.0	7.5×10^{-5}	5.1×10^{-3}	higher mRNA abundance in supraglottis
HHLA2	−1.1	7.7×10^{-5}	5.2×10^{-3}	higher mRNA abundance in supraglottis
C8orf85	−1.1	7.7×10^{-5}	5.2×10^{-3}	higher mRNA abundance in supraglottis
**Gene**	**Log2 Fold Change or Odds Ratio**	***p* Value**	**FDR**	**Mutation Difference**
DLEC1	−1.2	8.7×10^{-5}	5.6×10^{-3}	higher mRNA abundance in supraglottis
SGK2	−1.1	8.8×10^{-5}	5.6×10^{-3}	higher mRNA abundance in supraglottis
ALX4	−1.2	9.3×10^{-5}	5.8×10^{-3}	higher mRNA abundance in supraglottis
USH1C	−1.2	1.0×10^{-4}	6.1×10^{-3}	higher mRNA abundance in supraglottis
TUBA3C	−1.2	1.1×10^{-4}	6.6×10^{-3}	higher mRNA abundance in supraglottis
C17orf93	−1.2	1.1×10^{-4}	6.9×10^{-3}	higher mRNA abundance in supraglottis
CCL26	−1.0	1.2×10^{-4}	6.9×10^{-3}	higher mRNA abundance in supraglottis
OTX2	−1.1	1.2×10^{-4}	7.0×10^{-3}	higher mRNA abundance in supraglottis
STOX1	−1.0	1.2×10^{-4}	7.0×10^{-3}	higher mRNA abundance in supraglottis
OTOF	−1.1	1.3×10^{-4}	7.2×10^{-3}	higher mRNA abundance in supraglottis
LOC401387	−1.1	1.3×10^{-4}	7.6×10^{-3}	higher mRNA abundance in supraglottis
ZNF541	−1.1	1.3×10^{-4}	7.6×10^{-3}	higher mRNA abundance in supraglottis

AIPL1	−1.1	1.3×10^{-4}	7.6×10^{-3}	higher mRNA abundance in supraglottis
BHMT	−1.1	1.4×10^{-4}	7.7×10^{-3}	higher mRNA abundance in supraglottis
STRA8	−1.1	1.4×10^{-4}	7.7×10^{-3}	higher mRNA abundance in supraglottis
TMEM84	−1.1	1.4×10^{-4}	7.8×10^{-3}	higher mRNA abundance in supraglottis
RHBDL3	−1.0	1.5×10^{-4}	8.0×10^{-3}	higher mRNA abundance in supraglottis
B3GALT2	−1.0	1.5×10^{-4}	8.0×10^{-3}	higher mRNA abundance in supraglottis
MAB21L1	−1.0	1.5×10^{-4}	8.2×10^{-3}	higher mRNA abundance in supraglottis
FGA	−1.1	1.5×10^{-4}	8.3×10^{-3}	higher mRNA abundance in supraglottis
NPPC	−1.1	1.6×10^{-4}	8.6×10^{-3}	higher mRNA abundance in supraglottis
B3GALT1	−1.1	1.7×10^{-4}	8.8×10^{-3}	higher mRNA abundance in supraglottis
C11orf86	−1.1	1.7×10^{-4}	8.9×10^{-3}	higher mRNA abundance in supraglottis
ZIC1	−1.1	1.8×10^{-4}	9.1×10^{-3}	higher mRNA abundance in supraglottis
PRSS50	−1.1	1.8×10^{-4}	9.1×10^{-3}	higher mRNA abundance in supraglottis
SIGLEC16	−1.0	2.0×10^{-4}	9.7×10^{-3}	higher mRNA abundance in supraglottis
HKDC1	−1.0	2.0×10^{-4}	9.8×10^{-3}	higher mRNA abundance in supraglottis
NSD1	2.5×10^{-4}	2.5×10^{-4}	7.6×10^{-2}	more SNV mutations in supraglottis
COL11A1	3.8×10^{-4}	3.8×10^{-4}	7.6×10^{-2}	more SNV mutations in supraglottis
CTNNA2	1.1×10^{-3}	1.1×10^{-3}	7.6×10^{-2}	more SNV mutations in supraglottis
ERICH3	1.1×10^{-3}	1.1×10^{-3}	7.6×10^{-2}	more SNV mutations in supraglottis
FAM47C	1.1×10^{-3}	1.1×10^{-3}	7.6×10^{-2}	more SNV mutations in supraglottis
PKHD1L1	1.1×10^{-3}	1.1×10^{-3}	7.6×10^{-2}	more SNV mutations in supraglottis
LAMA2	1.5×10^{-3}	1.5×10^{-3}	8.5×10^{-2}	more SNV mutations in supraglottis
COL22A1	1.7×10^{-3}	1.7×10^{-3}	8.5×10^{-2}	more SNV mutations in supraglottis
ADCY2	2.5×10^{-3}	2.5×10^{-3}	9.6×10^{-2}	more SNV mutations in supraglottis
DCC	2.5×10^{-3}	2.5×10^{-3}	9.6×10^{-2}	more SNV mutations in supraglottis
**Gene**	**Log2 Fold Change or Odds Ratio**	***p* Value**	**FDR**	**Mutation Difference**
MUC16	2.9×10^{-3}	2.9×10^{-3}	9.6×10^{-2}	more SNV mutations in supraglottis
SI	3.3×10^{-3}	3.3×10^{-3}	9.6×10^{-2}	more SNV mutations in supraglottis
NPAP1	3.6×10^{-3}	3.6×10^{-3}	9.6×10^{-2}	more SNV mutations in supraglottis
LAMA1	3.6×10^{-3}	3.6×10^{-3}	9.6×10^{-2}	more SNV mutations in supraglottis
PCDH15	3.6×10^{-3}	3.6×10^{-3}	9.6×10^{-2}	more SNV mutations in supraglottis

Table S3. Pathway over-representations from genes with differential frequencies of SNVs and CNAs correlated with mRNAs between glottic and supraglottic LSCC. Blue indicates genes with higher mRNA abundance and copy number in the supraglottis, whereas red indicates genes with higher mRNA abundance and copy number in the glottis. The uncolored genes represent those with differences in SNV.

	**Pathway Identifier**	**R-HSA-397014**		
**1**	Description	#Entities total: 209 Muscle contraction		
	#Entities mapped in Reactome	17	Submitted entities found	ACTC1, ATP1A2, CACNG1, CACNG6, CASQ2, FGF12, KCNJ4, MYBPC2, MYH3, MYL4, MYLPF, NPPC, SLC8A2, SLN, TNNC2, TNNI1, TTN
	p-value	1.31×10^{-9}	FDR *q*-value	3.71×10^{-7}
**2**	Pathway identifier	R-HSA-390522		
	Description	#Entities total: 36 Striated Muscle Contraction		
	#Entities mapped in Reactome	7	Submitted entities found	ACTC1, MYBPC2, MYH3, MYL4, TNNC2, TNNI1, TTN
	p-value	3.05×10^{-7}	FDR *q*-value	4.33×10^{-5}
**3**	Pathway identifier	R-HSA-6809371		
	Description	#Entities total: 129 Formation of the cornified envelope		
	#Entities mapped in Reactome	11	Submitted entities found	KRT10, KRT2, KRT20, KRT34, LOC100653049, KRT37, KRT71, KRT84, LCE3D, LCE3E, SPRR2G
	p-value	7.77×10^{-7}	FDR *q*-value	7.36×10^{-5}
**4**	Pathway identifier	R-HSA-2672351		
	Description	#Entities total: 109 Stimuli-sensing channels		
	#Entities mapped in Reactome	8	Submitted entities found	ANO3, BEST2, BEST3, BSND, CASQ2, CLCNKA, SGK2, TRPM5
	p-value	7.77×10^{-5}	FDR *q*-value	0.00455
**5**	Pathway identifier	R-HSA-5576891		
	Description	#Entities total: 141 Cardiac conduction		
	#Entities mapped in Reactome	9	Submitted entities found	ATP1A2, CACNG1, CACNG6, CASQ2, FGF12, KCNJ4, NPPC, SLC8A2, SLN
	p-value	8.23×10^{-5}	FDR *q*-value	0.00455
**6**	Pathway identifier	R-HSA-6805567		
	Description	#Entities total: 214 Keratinization		
	#Entities mapped in Reactome	11	Submitted entities found	KRT10, KRT2, KRT20, KRT34, LOC100653049, KRT37, KRT71, KRT84, LCE3D, LCE3E, SPRR2G
	p-value	9.6×10^{-5}	FDR *q*-value	0.00455
**7**	Pathway identifier	R-HSA-983712		
	Description	#Entities total: 186 Ion channel transport		
	#Entities mapped in Reactome	10	Submitted entities found	ANO3, ATP1A2, BEST2, BEST3, BSND, CASQ2, CLCNKA, SGK2, SLN, TRPM5
	p-value	0.000139	FDR *q*-value	0.00565
**8**	Pathway identifier	R-HSA-112315		
	Description	#Entities total: 272 Transmission across Chemical Synapses		
	#Entities mapped in Reactome	12	Submitted entities found	ADCY2, ADCY8, CHRNA1, CHRNG, GRIK4, KCNJ4, PRKAG3, SLC1A2, SYN2, TUBA3C, TUBA3E, TUBAL3
	p-value	0.000196	FDR *q*-value	0.00697

**9**	Pathway identifier	R-HSA-112314		
	Description	#Entities total: 206 Neurotransmitter receptors and postsynaptic signal transmission		
	#Entities mapped in Reactome	10	Submitted entities found	ADCY2, ADCY8, CHRNA1, CHRNG, GRIK4, KCNJ4, PRKAG3, TUBA3C, TUBA3E, TUBAL3
	p-value	0.000319	FDR *q*-value	0.0101
**10**	Pathway identifier	R-HSA-9619483		
	Description	#Entities total: 29 Activation of AMPK downstream of NMDARs		
	#Entities mapped in Reactome	4	Submitted entities found	PRKAG3, TUBA3C, TUBA3E, TUBAL3
	p-value	0.000484	FDR *q*-value	0.0138
**11**	Pathway identifier	R-HSA-418990		
	Description	#Entities total: 33 Adherens junctions interactions		
	#Entities mapped in Reactome	4	Submitted entities found	CADM3, CDH12, CDH15, CDH18
	p-value	0.000802	FDR *q*-value	0.0202
**12**	Pathway identifier	R-HSA-112316		
	Description	#Entities total: 413 Neuronal System		
	#Entities mapped in Reactome	14	Submitted entities found	ADCY2, ADCY8, CHRNA1, CHRNG, DLGAP1, GRIK4, KCNJ4, KCNN1, PRKAG3, SLC1A2, SYN2, TUBA3C, TUBA3E, TUBAL3
	p-value	0.000851	FDR *q*-value	0.0202
**13**	Pathway identifier	R-HSA-2980736		
	Description	#Entities total: 90 Peptide hormone metabolism		
	#Entities mapped in Reactome	6	Submitted entities found	ADCY2, ADCY8, CHRNA1, CHRNG, DLGAP1, GRIK4, KCNJ4, KCNN1, PRKAG3, SLC1A2, SYN2, TUBA3C, TUBA3E, TUBAL3
	p-value	0.00104	FDR *q*-value	0.0228
**14**	Pathway identifier	R-HSA-5083625		
	Description	#Entities total: 17N Defective GALNT3 causes familial hyperphosphatemic tumoral calcinosis (HFTC)		
	#Entities mapped in Reactome	3	Submitted entities found	MUC16, MUC17, MUC6
	p-value	0.00124	FDR *q*-value	0.0233
**15**	Pathway identifier	R-HSA-5083636		
	Description	#Entities total: 17 Defective GALNT12 causes colorectal cancer 1 (CRCS1)		
	#Entities mapped in Reactome	3	Submitted entities found	MUC16, MUC17, MUC6
	p-value	0.00124	FDR *q*-value	0.0233
**16**	Pathway identifier	R-HSA-373080		
	Description	#Entities total: 95 Class B/2 (Secretin family receptors)		
	#Entities mapped in Reactome	6	Submitted entities found	GIP, PTH1R, SHH, WNT10B, WNT16, WNT8B
	p-value	0.00138	FDR *q*-value	0.0233
**17**	Pathway identifier	R-HSA-190840		
	Description	#Entities total: 18 Microtubule-dependent trafficking of connexons from Golgi to the plasma membrane		

	#Entities mapped in Reactome	3	Submitted entities found	TUBA3C, TUBA3E, TUBAL3
	p-value	0.00147	FDR *q*-value	0.0233
**18**	Pathway identifier	R-HSA-5083632		
	Description	#Entities total: 18 Defective C1GALT1C1 causes Tn polyagglutination syndrome (TNPS)		
	#Entities mapped in Reactome	3	Submitted entities found	MUC16, MUC17, MUC6
	p-value	0.00147	FDR *q*-value	0.0233
**19**	Pathway identifier	R-HSA-190872		
	Description	#Entities total: 19 Transport of connexons to the plasma membrane		
	#Entities mapped in Reactome	3	Submitted entities found	TUBA3C, TUBA3E, TUBAL3
	p-value	0.00173	FDR *q*-value	0.0259
**20**	Pathway identifier	R-HSA-389977		
	Description	#Entities total: 22 Post-chaperonin tubulin folding pathway		
	#Entities mapped in Reactome	3	Submitted entities found	TUBA3C, TUBA3E, TUBAL3
	p-value	0.00268	FDR *q*-value	0.0381
**21**	Pathway identifier	R-HSA-977068		
	Description	#Entities total: 24 Termination of O-glycan biosynthesis		
	#Entities mapped in Reactome	3	Submitted entities found	MUC16, MUC17, MUC6
	p-value	0.00345	FDR *q*-value	0.0468
**22**	Pathway identifier	R-HSA-438064		
	Description	#Entities total: 81 Post NMDA receptor activation events		
	#Entities mapped in Reactome	5	Submitted entities found	ADCY8, PRKAG3, TUBA3C, TUBA3E, TUBAL3
	p-value	0.00383	FDR *q*-value	0.0481
**23**	Pathway identifier	R-HSA-389960		
	Description	#Entities total: 25 Formation of tubulin folding intermediates by CCT/TriC		
	#Entities mapped in Reactome	3	Submitted entities found	TUBA3C, TUBA3E, TUBAL3
	p-value	0.00389	FDR *q*-value	0.0481

Table S4. RPPA differences between supraglottic and glottic LSCC. All results with *p* Value <0.05 are shown.

Protein/Phosphoprotein	*p* Value	FDR	Higher Protein Abundance in?
MIG6	2.6×10^{-4}	4.4×10^{-2}	Glottis
PREX1	3.7×10^{-4}	4.4×10^{-2}	Glottis
IRS1	1.1×10^{-3}	7.3×10^{-2}	Glottis
ETS1	1.3×10^{-3}	7.3×10^{-2}	Glottis
CHK1_pS296	1.5×10^{-3}	7.3×10^{-2}	Glottis
CKIT	5.9×10^{-3}	1.4 × 10−1	Glottis
ECADHERIN	6.4×10^{-3}	1.4 × 10−1	Supraglottis
HER3_pY1289	4.5×10^{-3}	1.4 × 10−1	Glottis
HSP70	5.9×10^{-3}	1.4 × 10−1	Glottis
PEA15_pS116	5.9×10^{-3}	1.4 × 10−1	Glottis
TRANSGLUTAMINASE	7.0×10^{-3}	1.4 × 10−1	Glottis
X1433BETA	5.4×10^{-3}	1.4 × 10−1	Glottis
S6_pS235S236	7.6×10^{-3}	1.4 × 10−1	Supraglottis
ACC_pS79	1.1×10^{-2}	1.9 × 10−1	Supraglottis
TIGAR	1.2×10^{-2}	2.0 × 10−1	Glottis
PDL1	1.4×10^{-2}	2.1 × 10−1	Glottis
RB_pS807S811	1.6×10^{-2}	2.1 × 10−1	Supraglottis
CABL	1.6×10^{-2}	2.1 × 10−1	Glottis
CHK2	1.9×10^{-2}	2.3 × 10−1	Supraglottis
DJ1	2.5×10^{-2}	2.3 × 10−1	Glottis
MRE11	2.3×10^{-2}	2.3 × 10−1	Glottis
P27	2.6×10^{-2}	2.3 × 10−1	Glottis
P38_pT180Y182	2.7×10^{-2}	2.3 × 10−1	Supraglottis
PEA15	2.5×10^{-2}	2.3 × 10−1	Glottis
PKCALPHA_pS657	2.4×10^{-2}	2.3 × 10−1	Glottis
S6_pS240S244	2.4×10^{-2}	2.3 × 10−1	Supraglottis
P27_pT198	2.1×10^{-2}	2.3 × 10−1	Glottis
TAZ	2.4×10^{-2}	2.3 × 10−1	Glottis
CK5	3.0×10^{-2}	2.4 × 10−1	Glottis
HER2	3.3×10^{-2}	2.6 × 10−1	Supraglottis
YB1_pS102	3.5×10^{-2}	2.6 × 10−1	Supraglottis
BAP1C4	3.6×10^{-2}	2.7 × 10−1	Supraglottis
ACVRL1	3.7×10^{-2}	2.7 × 10−1	Glottis
P62LCKLIGAND	4.0×10^{-2}	2.8 × 10−1	Supraglottis
NAPSINA	4.2×10^{-2}	2.8 × 10−1	Glottis
**Protein/Phosphoprotein**	***p* Value**	**FDR**	**Higher Protein Abundance in?**
RAB25	4.4×10^{-2}	2.9 × 10−1	Supraglottis
CYCLINE1	5.0×10^{-2}	3.1 × 10−1	Supraglottis
PI3KP110ALPHA	5.0×10^{-2}	3.1 × 10−1	Supraglottis
